# Supplementary material for: Unravelling reference bias in ancient DNA datasets
Source: Bioinformatics. 2024 Jul 3;40(7):btae436. doi: 10.1093/bioinformatics/btae436 (PMC11254355; doi:10.1093/bioinformatics/btae436)
Supplement: btae436_Supplementary_Data [file btae436_supplementary_data.zip › S1-S4_data_plots.zip!/S1-S4_data/elephant_sim_plots.zip!/elephant_sim_plots/verysensitive_bowtie_elephant_div0_50end_by_MQ.pdf]

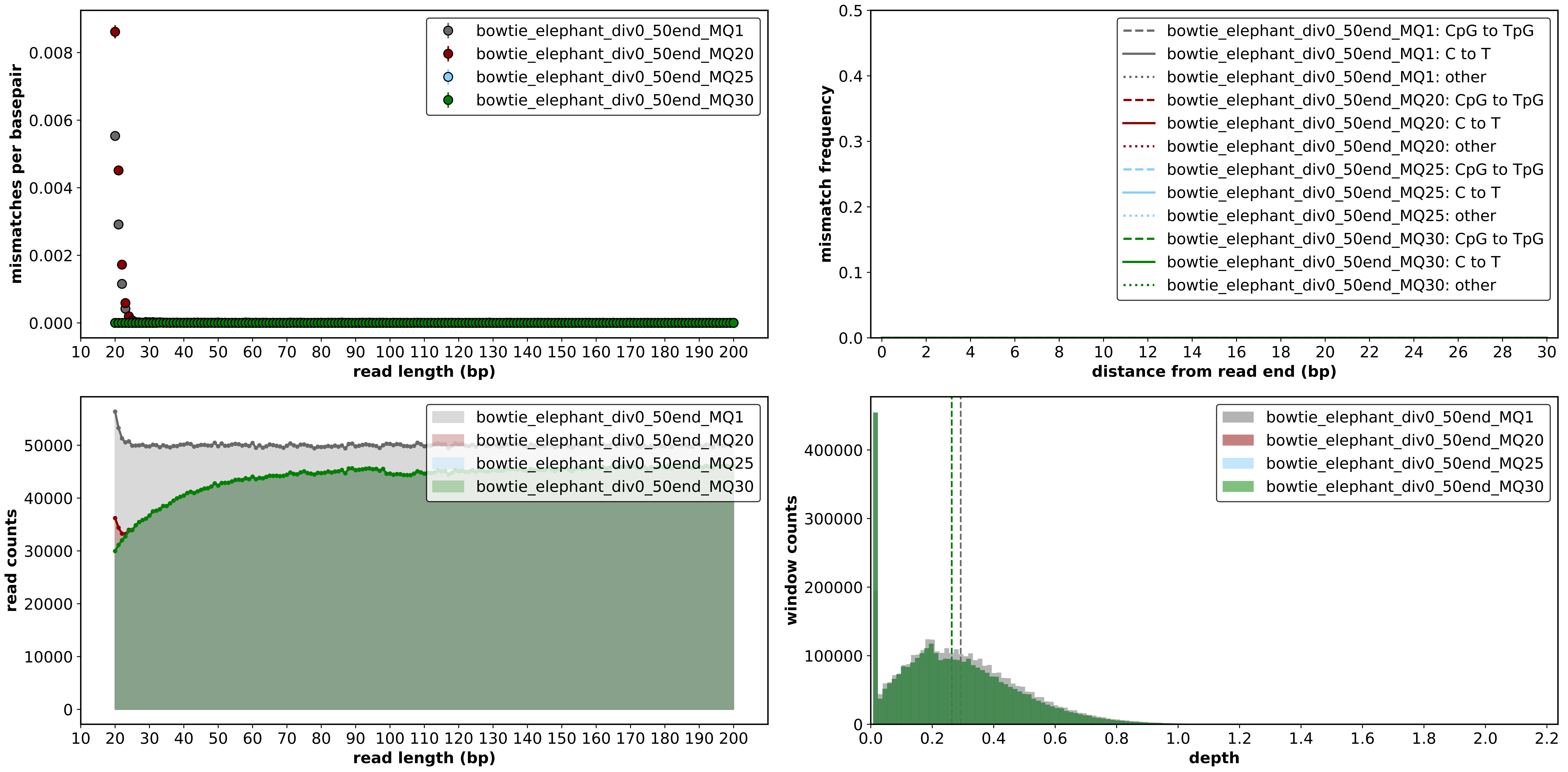

bowtie_elephant_div0_50end_MQ1
bowtie_elephant_div0_50end_MQ20
bowtie_elephant_div0_50end_MQ25
bowtie_elephant_div0_50end_MQ30
mismatches per basepair
0.000
0.002
0.004
0.006
0.008
read length (bp)
10 20 30 40 50 60 70 80 90 100 110 120 130 140 150 160 170 180 190 200
bowtie_elephant_div0_50end_MQ1: CpG to TpG
bowtie_elephant_div0_50end_MQ1: C to T
bowtie_elephant_div0_50end_MQ1: other
bowtie_elephant_div0_50end_MQ20: CpG to TpG
bowtie_elephant_div0_50end_MQ20: C to T
bowtie_elephant_div0_50end_MQ20: other
bowtie_elephant_div0_50end_MQ25: CpG to TpG
bowtie_elephant_div0_50end_MQ25: C to T
bowtie_elephant_div0_50end_MQ25: other
bowtie_elephant_div0_50end_MQ30: CpG to TpG
bowtie_elephant_div0_50end_MQ30: C to T
bowtie_elephant_div0_50end_MQ30: other
mismatch frequency
0.0
0.1
0.2
0.3
0.4
0.5
distance from read end (bp)
0 2 4 6 8 10 12 14 16 18 20 22 24 26 28 30
bowtie_elephant_div0_50end_MQ1
bowtie_elephant_div0_50end_MQ20
bowtie_elephant_div0_50end_MQ25
bowtie_elephant_div0_50end_MQ30
read counts
0
10000
20000
30000
40000
50000
read length (bp)
10 20 30 40 50 60 70 80 90 100 110 120 130 140 150 160 170 180 190 200
bowtie_elephant_div0_50end_MQ1
bowtie_elephant_div0_50end_MQ20
bowtie_elephant_div0_50end_MQ25
bowtie_elephant_div0_50end_MQ30
window counts
0
100000
200000
300000
400000
depth
0.0 0.2 0.4 0.6 0.8 1.0 1.2 1.4 1.6 1.8 2.0 2.2
